# Are ‘Endurance’ Alleles ‘Survival’ Alleles? Insights from the *ACTN3* R577X Polymorphism

**DOI:** 10.1371/journal.pone.0017558

**Published:** 2011-03-03

**Authors:** Carmen Fiuza-Luces, Jonatan R. Ruiz, Gabriel Rodríguez-Romo, Catalina Santiago, Félix Gómez-Gallego, Thomas Yvert, Amalia Cano-Nieto, Nuria Garatachea, María Morán, Alejandro Lucia

**Affiliations:** 1 Universidad Europea de Madrid, Madrid, Spain; 2 Department of Physical Education, School of Physical Activity and Sport Sciences, University of Granada, Granada, Spain; 3 Department of Biosciences and Nutrition at NOVUM, Unit for Preventive Nutrition, Karolinska Institutet, Stockholm, Sweden; 4 INEF, Universidad Politécnica de Madrid, Madrid, Spain; 5 SUAP Archena, Hospital Morales Meseguer, Murcia, Spain; 6 Faculty of Health and Sport Science, Department of Physiotherapy and Nursing, University of Zaragoza, Zaragoza, Spain; 7 Centro de Investigación Hospital 12 de Octubre and CIBERER, Madrid, Spain; Pennington Biomedical Research Center, United States of America

## Abstract

Exercise phenotypes have played a key role for ensuring survival over human evolution. We speculated that some genetic variants that influence exercise phenotypes could be associated with exceptional survival (i.e. reaching ≥100years of age). Owing to its effects on muscle structure/function, a potential candidate is the Arg(R)577Ter(X) polymorphism (rs1815739) in *ACTN3*, the structural gene encoding the skeletal muscle protein α-actinin-3. We compared the *ACTN3* R577X genotype/allele frequencies between the following groups of ethnically-matched (Spanish) individuals: centenarians (*cases*, n = 64; 57 female; age range: 100–108 years), young healthy controls (n = 283, 67 females, 216 males; 21±2 years), and humans who are at the two end-points of exercise capacity phenotypes, i.e. muscle endurance (50 male professional road cyclists) and muscle power (63 male jumpers/sprinters). Although there were no differences in genotype/allele frequencies between centenarians (RR:28.8%; RX:47.5%; XX:23.7%), and controls (RR:31.8%; RX:49.8%; XX:18.4%) or endurance athletes (RR:28.0%; RX:46%; XX:26.0%), we observed a significantly higher frequency of the X allele (P = 0.019) and XX genotype (P = 0.011) in centenarians compared with power athletes (RR:47.6%; RX:36.5%;XX:15.9%). Notably, the frequency of the null XX (α-actinin-3 deficient) genotype in centenarians was the highest ever reported in non-athletic Caucasian populations. In conclusion, despite there were no significant differences with the younger, control population, overall the *ACTN3* genotype of centenarians resembles that of world-class elite endurance athletes and differs from that of elite power athletes. Our preliminary data would suggest a certain ‘survival’ advantage brought about by α-actinin-3 deficiency and the ‘endurance’/oxidative muscle phenotype that is commonly associated with this condition.

## Introduction

Identifying candidate gene variants associated with ‘longevity assurance’ is possible by studying the genotype of centenarians [1]. This group of people are the survival tail of population; indeed, they escaped diseases of the pre-antibiotic era, and have postponed/avoided several ageing-related diseases as well as their fatal consequences [2]. Genetically we are still citizens of the Paleolithic era living in the 21st century [3], [4]. In the Paleolitithic societies survival was essentially linked to exercise (e.g. ‘long time tracking persistence’ for successful hunting) [5], [6]. Therefore, it would be plausible to speculate that some identified genetic variants associated with exercise phenotypes could also be associated with exceptional survival.

The *ACTN3* gene encodes for the synthesis of α-actinin-3 in skeletal-muscle fibres, a sarcomeric protein necessary for producing ‘explosive’ powerful contractions. A premature stop codon polymorphism [Arg(R)577Ter(X), rs1815739] in *ACTN3* was first described by North et al. [7]. This genetic variation, which probably preceded the appearance of anatomically modern humans in Europe and Asia (∼40.000–60.000 years ago) can affect exercise phenotypes [8]. The α-actinin-3 deficient XX genotype (with a frequency of ∼18% among European Caucasians) is believed to preclude top-level athletic performance in ‘pure’ power and sprint sports (e.g. sprinting, jumping events), especially in women [9]. In contrast, compared with the general population, the X allele tends to be overrepresented in those humans with an ‘extreme endurance phenotype’, i.e. elite endurance athletes [9], [10]. Mechanistic explanation for the latter finding might be found in the α-actinin-3 knockout (KO) mouse developed by Prof. North's group. Compared with wild-type mice, the muscles of the KO mouse exhibit 33% higher endurance and a shift towards increased activity of mitochondrial oxidative metabolism [8], [11]. With regards to this, improved oxidative pathways in mitochondria may be a common factor linking physical fitness and decreased disease risk [12], [13]. For the abovementioned reasons, it could be speculated that the shift towards more efficient aerobic metabolism associated with the XX genotype might confer a ‘survival advantage’.

The main purpose of the present study was to compare the allelic and genotypic frequency of the *ACTN3* R577X polymorphism between Spanish centenarians (aged 100–108 years), and a group of healthy young adults (controls, aged <40 years) of the same ethnic origin. A secondary aim was to compare the data obtained in centenarians with those of two groups of humans who show the two ‘extreme’ phenotypes of the exercise performance continuum, i.e. elite (world-class) athletes excelling in endurance and power sports respectively. We hypothesized that: (i) the XX genotype tends to be overrepresented in centenarians compared with the general population; (ii) the *ACTN3* R577X allelic/genotypic frequency distribution of centenarians is similar to that of endurance athletes; and (iii) the XX genotype will be overrepresented in centenarians compared with the power athlete group.

## Methods

The study was designed and carried out in accordance with the recommendations for the human genotype-phenotype association studies recently published by the *NCI-NHGRI Working Group on Replication in Association Studies* [14]. These recommendations include among others, the following items: indicating time period and location of subject recruitment, success rate for DNA acquisition, sample tracking methods, and genotyping with a second technology in a second laboratory.

### Participants

Written consent was obtained from each participant. The study protocol was approved by the institutional ethics committee (*Universidad Europea de Madrid* (UEM). Spain) and was in accordance with the Declaration of Helsinki for Human Research of 1974 (last modified in 2000).

All the study participants were of the same Caucasian (Spanish) descent for ≥3 generations. The majority (∼90%) of them lived most of their lives and were born in the same areas of Spain (*Meseta Castellana, ∼600 m altitude*).

### Young adults (controls)

Inclusion criteria for this group were (i) to be woman or a man, aged ≤40 years, (ii) free of any diagnosed cardiometabolic disease, (iii) having no known family history of high longevity (90+ years). During year 2008, we extracted genomic DNA from saliva samples of 283 University students of both genders (67 females, 216 males; mean±SD age: 21±2 years) who met the aforementioned criteria and were randomly selected from two Universities of the same city (Madrid, Spain): *Universidad Politécnica* (N = 200) and UEM (n = 83). Their maximal oxygen uptake (VO_2max_) was 48.0±4.3 ml·kg^−1^·min^−1^. These controls served as subjects in previous research from our group [15], [16].

### Centenarians (cases)

During 2009–2010 we obtained DNA from saliva samples in 64 centenarians of both genders (57 female, 7 male; 102±1 years, range: 100, 108) living in nursing residencies of the Spain central area (*Meseta Castellana*). The most prevalent diseases in the centenarians' cohort were osteoarthritis (72%), hypertension (62.5%), dementia (50%) and coronary artery disease (30%). Three centenarians were free of any diagnosed disease.

### Elite endurance athletes

This sample included the best 50 professional male Spanish (26±3 years) road cyclists in the last years, i.e. all Tour de France finishers one or more time, including stage winners and top-3 finishers [10]. Their VO_2max_ was 73.1±6.2 ml·kg^−1^·min^−1^.

### Elite power athletes

This sample included 63 elite power (track and field) Spanish male athletes (27±3 years), including the best Spanish jumpers (n = 18) and sprinters in recent years (n = 45). Thirteen of them were Olympians during the period 2000–2008. Their VO_2max_ was 60.3±5.5 ml·kg^−1^·min^−1^.

### Genotype assessment of the ACTN3 R577X polymorphism

Genotyping was performed specifically for research purposes. The researchers in charge of genotyping were totally blinded to the participants' identities, i.e. saliva samples were tracked solely with bar-coding and personal identities were only made available to the main study researcher who was not involved in actual genotyping.

### ‘Reference’ genotyping

Genotyping was originally performed during 2008–2010 in the genetics laboratory of UEM (Madrid) following previously described methodology [10]. The polymerase chain reaction (PCR) was performed in order to amplify the sequence containing the mutation. A fragment of 291 bp was amplified with the following primers: ACTN3-F 5′- CTGTTGCCTGTGGTAAGTGGG labelled a 5′ with VIC and ACTN3-R 5′- TGGTCACAGTATGCAGGAGGG. The PCR conditions were as follows: initial denaturing at 95°C 5 min; 35 cycles at 95°C 30 s, 60°C 30 s, 72°C 30 s and a final extension at 72°C 10 min. *ACTN3* genotypes were established by enzymatic digestion of amplicons with *Dde I*. The R577X change creates a restriction site resulting in fragments of 108, 97 and 86 bp. Digestion of the R577 allele results in fragments of 205 and 86 bp, and digestion of the 577X allele results in fragments of 108, 97 and 86 bp. The digestion products detected by capillary electrophoresis (ABI Prism 310 genetic analyzer; Applied Biosystems, Foster City, CA) were those labelled with VIC, i.e. 108 bp for 577X, and 205 bp for R577.

### Reliability assessment of genotype analysis in a second laboratory

Following recent recommendations [14], genotype results of the *ACTN3* R577X polymorphism were replicated in a different laboratory (*Progenika Biopharma, Parque Tecnológico de Zamudio*, Derio-Vizcaya, Spain) using a different technology, i.e. newly developed low-density DNA microarray based on allele-specific probes. The design, fabrication, validation and analysis of the arrays were performed following the procedure described elsewhere [17]. In brief, the PCR products were fluorescently labelled and hybridized to the DNA microarray in an automated platform (Ventana Medical Systems, Inc., Tucson, AZ, USA). The microarrays were scanned (Innopsys S.A., Carbonne, France) and we determined variants using a developed software that converts the intensity of the spots into the genotype of each variant.

### Statistical analysis

We tested Hardy-Weinberg equilibrium using a χ^2^ test. All statistical analyses were performed using the PASW (v. 18.0 for WINDOWS, Chicago). Genotype/allele frequencies were compared among the four study groups (centenarians, controls, endurance and power athletes) using a χ^2^ test with α set at 0.05. We used logistic regression analysis to analyse the association between alleles and longevity after adjusting for sex.

## Results

There were no failures in sample collection, DNA acquisition or genotyping procedures, except for 5 centenarians, for which the amount of DNA gathered from saliva was insufficient to allow *ACTN3* R577X genotype assessment. Parallel genotyping results showed 100% concordance between the two laboratories.

**Figure 1:**
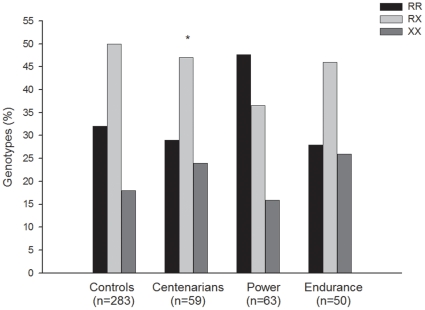
Genotype distribution of the *ACTN3* R577X polymorphism in our study cohorts. Symbol: *P = 0.011 for centenarians vs. power athletes.

**Figure 2:**
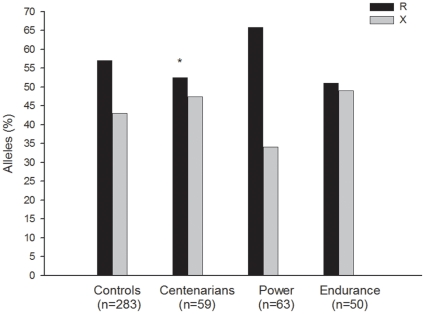
Allele distribution of the *ACTN3* R577X polymorphism in our study cohorts. Symbol: *P = 0.019 for centenarians vs. power athletes.

Genotype distributions met Hardy-Weinberg equilibrium in all study groups (all P>0.1). Figures 1 and 2 show the genotype and allele frequencies respectively in the different study groups. The *ACTN3* R577X genotype distributions in our healthy controls were overall similar to those previously reported in healthy Spanish adults [10] and European Caucasians, i.e. ∼18% frequency of the XX genotype [7]. There were no differences in genotype or allele frequencies between centenarians, and controls or endurance athletes (all P>0.1). We repeated the analyses by comparing X carriers vs. non-X carriers, or R carriers and non-R carriers and found no significant differences between groups (all P>0.1). However, we observed significant differences in genotype and allele frequencies between centenarians and power athletes: the frequency of X carriers was higher in the centenarians compared with the power athletes group (Figure 1, P = 0.011), and the frequency of the X allele was also higher in centenarians than in power athletes (Figure 2, P = 0.019).

The odds ratio (OR) of being a centenarian if the subject had the X allele was not significant: 1.236 (95% confidence interval (CI): 0.484–1.803), 1.041 (95% CI: 0.486–2.230), and 1.678 (95% CI: 0.928–3.033) compared to the control, endurance athletes, and power athletes group, respectively, after adjusting for sex. Likewise, the OR of being a centenarian if the subject had the XX genotype did not reach statistical significance: 1.032 (95% confidence interval (CI): 0.863–1.234), 1.132 (95% CI: 0.784–1.636), and 1.165 (95% CI: 0.945–1.437) compared to the control, endurance athletes, and power athletes group, respectively, after adjusting for sex.

## Discussion

Although the allelic/genotypic frequency of the *ACTN3* R577X polymorphism did not significantly differ between centenarians and the control population, a major finding of our study was that overall, the allelic/genotypic distribution of the *ACTN3* R577X polymorphism in centenarians was closest to that of those humans who arguably show the most ‘extreme’ muscle endurance phenotypes, i.e. professional road cyclists able to excel in such demanding events as the Tour de France [10]. Notably, the frequency of the null XX genotype in our cohort of centenarians (24%) was the highest ever reported in non-athletic Caucasian populations, and is only surpassed by the values of the professional road cyclists we studied (26%) and the elite endurance athlete's cohort (made up of various types of Olympic-class endurance athletes, included road cyclists) of a previous report by Yang *et al* on Australian athletes (30%) [9].

Though natural selection in the human genome accelerated during the past 40,000 years, probably less than 1% of our genes arose during the late Pleistocene and Holocene epochs (126,000–8,000 years before present), that is, after the Paleolithic era [4]. Genetically, therefore, we are still citizens of the Paleolithic era living in the 21st century; our genetic makeup is shaped to support the balance between energy intake and energy expenditure that was common to Paleolithic hunter–gatherer societies [3]. Among these people, survival depended upon the obligatory link between energy intake (food consumption) and energy expenditure for ensuring food acquisition [18]. Thus, ‘long time tracking persistence’ (i.e. mostly endurance or ‘aerobic’ exercise, such as walking/running 20 or more km/day) was likely necessary for successful hunting and thus for survival [5], [6]. In this context, genetic variants with a documented influence on exercise/muscle related phenotypes could also be associated with survival. For instance, the D allele of the 287 bp Ins(I)/Del(D) polymorphism [rs1799752] in intron 16 of the angiotensin converting enzyme (*ACE*) gene, which has been associated with several exercise phenotypes, e.g. preservation of muscle mass/strength, included in people aged 60 years and over [19], seems to be more frequent in some cohorts of European centenarians compared with their younger ethnically-matched referents [20], [21], [22]. Here, we hypothesized that the *ACTN*3 R577X variation is a strong candidate to influence survival over human evolution. Indeed, among the numerous genes that contribute to the ‘fitness’ or ‘performance’ phenotype, the *ACTN*3 is the first structural skeletal muscle gene for which such an association has been demonstrated [23]. The propagation of *ACTN3* deficiency in humans with marked variations in X-allele frequencies among different ethnic groups [7], [24] has raised the possibility that the X allele has been positively selected in some populations. Owing to its effect on skeletal muscle metabolism (see below), the X allele probably provided some sort of fitness advantage to modern humans adapting to the novel Eurasian environment [25].

Compared with wild-type mice, the muscles of the α-actinin-3 deficient mouse exhibit 33% higher endurance (during treadmill running) and a shift towards more oxidative pathways of metabolism, i.e. increased activity of mitochondrial oxidative metabolism [11], [25]. The XX genotype shows especially high frequency (26%) in professional road cyclists, who exhibit extremely high values of aerobic capacity and muscle efficiency. The high muscle efficiency of these athletes is attributable to a higher contribution of slow (type I) muscle type fibers compared with the less efficient type II (‘anaerobic’) subtype, in which α-actinin-3 is almost exclusively expressed [26]. In addition, a shift towards more oxidative muscle phenotypes associated with the XX genotype could have a beneficial influence on health related phenotypes. Classic studies in rats by Koch and Britton's group showed that improved oxidative pathways in mitochondria may be a common factor linking physical fitness and decreased disease risk [12], [13]. In humans, the metabolic effects of *ACTN3* genotype may act as a modifier of disease severity: the X allele has recently been shown to benefit exercise capacity phenotypes (i.e. aerobic capacity) in women with muscle (McArdle) disease [27]. On the other hand, there appears to be an evolutionary predetermined ‘trade-off’achieved over human evolution through the maintenance of genetic variation by balancing natural selection in performance traits for power vs. more endurance oriented exercise tasks [28], [29]. That is, an individual is inherently predisposed towards being more of a ‘sprinter’or a ‘stayer’ [23]. With regards to this, those genotypes associated with the latter type of phenotype (e.g. the XX genotype) might have provided a certain survival benefit over human evolution as opposed to other animals for which the ability to produce ‘explosive’ muscle actions (rapid accelerations and decelerations) is vital for survival, i.e. predator and prey quadrupeds [30].

Published data on *ACTN3* genotypes and muscle phenotypes with regards to ageing are controversial. Compared with the age-matched wild type mouse, the ageing KO mouse shows greater loss of fast muscle force generation and male muscle mass, good maintenance of grip strength, and increased oxidative metabolism and greater force recovery after fatigue [31]. San Juan et al. [32] observed no association between *ACTN3* genotypes and functional capacity and muscle phenotypes (included 1RM leg press) in women aged 61–80 years; whereas others found that (i) in adults with a mean age ∼65 years, the XX genotype was associated with higher knee extensor concentric peak power compared with RR/RX genotypes, especially in women [33], and (ii) in women (aged ∼60 years on average) the XX genotype was associated with lower peak torque in knee extensor muscles [34]. Recently, we observed no major influence of *ACTN3 genotypes* on muscle phenotypes (leg and grip strength, walking and stair climbing ability) in nonagenarians [35]. Differences in the age, baseline physical capacity and ethnic origin make comparison between studies difficult. Assessing the putative effects of *ACTN3* genotypes on the muscle phenotypes of centenarians is nevertheless a very difficult task: the expected poor physical condition and lack of independence in most of them (which at least was the case in our cohort) precludes the use of valid tools for phenotype assessment in this population.

We believe that a strength from our study comes from our group of cases, i.e. being all centenarians. Indeed, a well accepted approach for identifying those genetic factors that are associated with ‘longevity assurance’ is to study the genotype of centenarians. These people are the survival tail of population, and living 100 or more years is a rare phenotype, i.e. ≤1 every 10,000 people [1], [2]. However, our results should be taken with caution due to the relatively low number of centenarians we studied. Indeed, a sample size of ∼400 centenarians would have been needed in order to reach sufficient statistical power (≥0.8). To note is that we only included males in the two athletic cohorts due to the higher competition success (at the international level) of Spanish male athletes compared to their women counterparts. This ensured that both cohorts were really in both end-points of the human performance continuum. With regards to this, it must be kept in mind that the X allele seems to be more influential to elite athletic status in women than in men [9]. It thus remains to be determined if our results can be corroborated with women's athletic cohorts. Future studies are also necessary with larger samples of centenarian males in order to replicate the findings we obtained with our cohort of centenarians, which was mainly composed of women. On the other hand, longevity is likely a complex, polygenic trait, which could be influenced by numerous gene-environment and gene-gene interactions, including those interactions between genetic variants that might not influence longevity *individually*.

In summary, although we did not find significant differences with the younger, control population, overall the *ACTN3* genotype profile of centenarians resembles that of world-class elite endurance athletes and seems to differ from that of elite power athletes. Our preliminary data would suggest a certain ‘survival’ advantage brought about by α-actinin-3 deficiency and the ‘endurance’/oxidative muscle phenotype that is commonly associated with this condition; yet, more data on larger cohorts and on other ethnicities are necessary. Despite its uniqueness, the ‘oldest old’ is the most rapidly growing age group in western countries [36]. It is also the least physically active, and that generating the highest healthcare expenditures, largely due to loss of physical function [37]. Thus, identifying those genetic variants that are associated with exceptional longevity and rate of decline of physical function at the end of the human lifespan is of potential medical relevance. Our results might provide insights into the putative role on human survival of some genetic polymorphisms which are known to influence muscle function, of which the *ACTN3* R577X is currently the best example.
